# Children’s oral health: Are dentists and physicians prepared enough? A cross-sectional interdisciplinary survey carried out in North Italy

**DOI:** 10.1007/s40368-026-01203-0

**Published:** 2026-04-01

**Authors:** M. G. Cagetti, C. Salerno, S. Cirio, G. Campus

**Affiliations:** 1 University of Milan, Milan, Italy; 2 University of Gothenburg, Gothenburg, Sweden; 3 Department of Cariology, Saveetha Dental Collage and Hospitals, Chennai, India

**Keywords:** Child oral health, Interdisciplinary survey, Knowledge, Education

## Abstract

**Purpose:**

Physicians and dentists have a central role in preventing and managing oral health problems in children. This survey aimed to evaluate their knowledge and attitudes towards paediatric oral health.

**Methods:**

A cross-sectional survey was conducted via an anonymous questionnaire distributed to healthcare professionals in the province of Milan, Italy, assessing knowledge of paediatric dentistry and exploring attitudes towards clinical advice for improving or maintaining children’s oral health. Descriptive statistics were calculated for all items; the total number of correct answers served as a measure of participants’ preparedness. Comparisons were conducted using Pearson’s Chi-squared test or Fisher’s exact test and ANOVA, followed by Tukey’s post hoc test.

**Results:**

Overall, 843 healthcare professionals participated (424 physicians and 419 dentists). Comparison between physicians and dentists revealed differences in knowledge and attitudes, with dentists achieving higher scores (*p* < 0.01), although variability in responses was evident in both groups. Dentists demonstrated superior knowledge concerning the number of primary teeth (99.52% vs. 81.13%, *p* < 0.01) and the effectiveness of sealants (96.9% vs. 82.08%, *p* < 0.01), whilst physicians recognised that toothpaste with 500 ppm *F* was not suitable for all children (59.91% vs. 57.28%, *p* = 0.48) and understood that initial caries lesions can be reversed (86.79% vs. 66.83%, *p* < 0.01). ANOVA revealed variability across health professionals with different specialties (*F*-statistic: 9.59 for physicians, 12.40 for dentists; *p* < 0.01).

**Conclusion:**

Dentists showed greater preparedness than physicians; however, variability remained, partly attributable to inconsistent guidelines, underscoring the need for targeted educational and harmonised recommendations.

**Supplementary Information:**

The online version contains supplementary material available at 10.1007/s40368-026-01203-0.

## Introduction

The prevention of long-term oral health issues, the promotion of lifelong healthy habits, and the support of proper oral–facial development rely on compliance with preventive measures and early oral care. General dentists often encounter young patients and are expected to provide appropriate care or, when necessary, refer them to paediatric dental specialists. Similarly, physicians are involved in assessing a patient’s risk of developing oral health issues and providing guidance to parents and children on how to prevent them. They can also offer timely referrals and advise when oral healthcare is necessary. This proactive approach helps to avoid sudden episodes of pain and infection that might otherwise require emergency treatment or extensive restorative procedures, which are often performed on young patients under sedation or general anaesthesia. Effective coordination between healthcare professionals is essential to ensure optimal care for young patients (Pierce et al. 2002; Dela Cruz et al. 2004; McQuistan et al. 2006).

A lack of knowledge amongst physicians regarding child oral health may hinder their ability to provide accurate advice on nutrition, preventive care, or the importance of early dental visits (Douglass et al. 2009). General dentists may also face difficulties when treating children, particularly in cases that require specialised dental treatments or advanced behavioural management strategies (Kohli et al. 2022). These gaps in knowledge can result in suboptimal treatments, delayed diagnoses, and unmet dental needs (Braun and Cusick 2016). The well-documented connection between oral and systemic health underscores the need for strong interdisciplinary collaboration between dentists and physicians in managing paediatric patients (Casamassimo 2000; Zerman et al. 2024). Oral health issues in children can contribute to systemic problems such as poor nutrition, impaired growth, and a heightened risk of systemic infection (Ferrazzano et al. 2020). Consequently, ensuring children’s overall health relies on the comprehensive training and knowledge of both physicians and dentists in various aspects of oral and general health.

Training in paediatric dentistry should be an integral part of university education for healthcare professionals in Italy, as in all countries. However, the level of knowledge imparted to physicians and dentists in this field can vary significantly. While medical and dental students may encounter paediatric patients during their clinical training, the depth and scope of knowledge and practical experience in oral problems differ greatly between the two disciplines and even across universities offering the same course. Although the European Academy of Paediatric Dentistry developed Curriculum Guidelines for Education and Training in Paediatric Dentistry in 2007 (EAPD 2009), the document has not been updated, and no recent standardised Core Curriculum has been established (Field et al. 2025). This lack of an updated framework highlights the need for a contemporary, unified approach to ensure consistent training and competence in paediatric dentistry across Europe. Therefore, identifying potential gaps in knowledge and training amongst both dentists and physicians is crucial. Investigating any gaps in this area would be helpful in improving education, promoting an interdisciplinary approach to paediatric care, and ultimately ensuring comprehensive and appropriate healthcare for children.

The lack of interaction between physicians and dentists is not always attributable to insufficient knowledge of a particular topic. In some cases, the recommendations provided to parents may vary significantly between specialists, reflecting differing perspectives on the same issue and sometimes conflicting guidelines issued from their respective disciplines. One notable example is the varying recommendations regarding the duration of breastfeeding. For instance, whilst organisations like the World Health Organization (WHO) advocate for breastfeeding up to 2 years or beyond (WHO 2025), the American Academy of Pediatric Dentistry recommends it up to 12 months (AAPD 2008). These differences highlight a lack of consensus and unified communication across disciplines, which can undermine patient trust and adherence to advice. These inconsistencies may result in confusion among parents and caregivers, ultimately undermining trust in healthcare providers.

There are few studies investigating the knowledge of general dentists, physicians, and paediatricians on paediatric dentistry, and these reflect the limited interaction between these specialties (Di Giuseppe et al. 2006; Nammalwar and Rangeeth 2012; Aburahima et al. 2020; Dickson-Swift et al. 2020; Lolo et al. 2021; Farsi and Alagili 2023; Tadin and Dzaja 2023). Amongst these, only one study, conducted in Italy in 2006, examined paediatricians’ knowledge of oral disease prevention (Di Giuseppe et al. 2006). No previous study has directly compared the knowledge of different specialists to determine whether there is a shared understanding that could enhance communication and support collaborative care. This interdisciplinary survey aims to evaluate the knowledge and attitudes of dentists and physicians from various disciplines regarding paediatric dentistry. The null hypothesis asserts that no differences exist in the level of knowledge and attitudes amongst both general and specialist medical and dental practitioners.

## Materials and methods

The present study was designed as an observational, questionnaire-based, cross-sectional survey, in compliance with the Declaration of Helsinki and approved by the Ethics Committee of the University of Sassari, Sassari, Italy (No. AOU_SS 386 on 22-03-2022). The reporting of this study follows the STROBE (Strengthening the Reporting of Observational Studies in Epidemiology) guidelines for cross-sectional studies.

An anonymous questionnaire based on a previously validated tool (Tadin and Dzaja 2023) consisting of 17 closed-ended, multiple-choice or dichotomous questions was used (Supplementary file S1). The quantitative analysis of the questionnaire’s accuracy was conducted by submitting it to 12 experts, including 3 paediatric dentists, 3 general dentists, 3 paediatricians, and 3 family physicians. The content validity of each item was assessed using the content validity index (CVI) and the content validity ratio (CVR) (Polit et al. 2007). The content validity index of the scale (S-CVI) was calculated using the universal agreement method. Based on expert opinions, the S-CVI for the entire instrument was 1.00, and the S-CVR was 0.98 (Supplementary file S2).

The questionnaire was pre-tested in October 2022 for comprehensibility on a small sample of 15 dentists and 15 physicians not included in the survey. After completing the questionnaire, participants were contacted to assess whether they had encountered any difficulties in understanding the questions. They were given a comprehension score ranging from 1 (extreme difficulty) to 5 (no difficulty), with a final score of 4.35 ± 0.22 obtained.

The questionnaire included two questions related to the demographic characteristics of the sample (medical specialisation field and years of professional experience), nine questions assessing basic knowledge of paediatric dentistry, and six questions exploring attitudes of dentists and physicians regarding clinical advice for improving or maintaining children’s oral health. An online version of the anonymous questionnaire was created using Google Form (Google LLC, Mountain View, CA, USA) and distributed via email. Before the first question, a brief description of the study’s purpose was provided, and participants were asked to sign an online informed consent form. If consent was not given, the questionnaire was automatically closed.

Physicians and dentists were contacted using the email addresses provided by the Italian Federation of Doctors and Dentists in the province of Milan, Italy, as all licensed professionals in the country are required to provide an email address. Non-respondents received a follow-up invitation 2 months later. The province of Milan covers an area of 1575 km^2^ and is highly urbanised, with a population density exceeding 2000 inhabitants per km^2^, making it the third most densely populated province in Italy. Approximately 27,000 healthcare professionals are registered with the Italian Federation of Doctors and Dentists in the province, including about 4000 dentists.

To determine the sample size, an a priori power analysis was conducted. Assuming an expected frequency of 50%, a statistical power of 99.00%, an alpha error of 5%, and a design effect of 1, the minimum required sample size was calculated to be 648 participants.

The survey was carried out from January 2023 to December 2023, with data downloaded in January 2024.

### Statistical analysis

Data were downloaded from the survey platform, imported into a Microsoft Excel (Microsoft Corporation, WA, USA) spreadsheet, and quality checked by one of the authors to ensure accuracy. Questionnaires with incomplete responses were excluded from the analysis. Statistical analyses were performed using STATA® 18.0 SE (StataCorp LLC, TX, USA). Fourteen questions had definitive correct answers. One additional question, “Up to what age should breastfeeding be recommended?”, lacked a single consensus answer supported by the scientific community. Nonetheless, this question was included to explore differing perspectives between dentists and physicians. Descriptive statistics were calculated for all items to provide an overview of the results. Discrete variables were presented as absolute and relative frequencies (%), and comparisons between groups (dentists and physicians) were conducted using Pearson’s Chi-squared test or Fisher’s exact test, as appropriate. In addition to *p*-values, effect sizes were calculated to assess the magnitude of observed differences. Cohen’s d was used for continuous variables (total score), and Cramér’s V was calculated for selected categorical comparisons. Effect sizes were interpreted according to conventional benchmarks (small = 0.2, medium = 0.5, large = 0.8 for Cohen’s *d*). The total number of correct answers was used as a measure of participants’ preparedness on the topic, with a maximum possible score of 14 points. Scores (continuous outcome) were presented as mean and standard deviation. Comparisons of scores between groups, according to the type of specialisation in each group (physicians or dentists), were performed using ANOVA, followed by Tukey’s post hoc test. Multivariable linear regression was conducted to investigate the relationship between sociodemographic variables and the total knowledge score. For these models (using STATA’s regress command), sociodemographic variables (group: physicians or dentists; years of working experience) were treated as explanatory variables, and the total score (ranging from 0 to 14) served as the dependent variable. To ensure robustness, multicollinearity was assessed using the Belsley–Kuh–Welsch technique, while heteroskedasticity and normality of residuals were evaluated with the White test and the Shapiro–Wilk test, respectively. Interaction effects were examined using the likelihood ratio test. Alpha error was set at 5%.

## Results

A total of 1023 health professionals accessed the online questionnaire, with 843 completing it, comprising 424 physicians and 419 dentists. This resulted in an overall response rate of just over 3% (Table 1). A large proportion of physicians (58.96%) and dentists (49.16%) reported having over 20 years of professional experience, indicating a workforce largely composed of seasoned practitioners. Among physicians, paediatricians and those in other medical specialties have the highest proportion of long-tenured practitioners, whilst general/family physicians exhibit a more balanced distribution of experience levels. Amongst dentists, paediatric dentists and orthodontists tend to have a greater proportion of less experienced practitioners compared to other dental specialties, suggesting potential growth in these fields.

**Table 1:** Demographic characteristics of the sample included in the survey

	Work experience				
	< 5 years	5–10 years	11–15 years	16–20 years	> 20 years
	*N* (%)	*N* (%)	*N* (%)	*N* (%)	*N* (%)
Specialisation					
Physicians					
Paediatricians (*n* = 171)	13 (7.60)	25 (14.62)	19 (11.11)	13 (7.60)	101 (59.06)
General/family physicians (*n* = 49)	12 (24.49)	3 (6.12)	7 (14.29)	3 (6.12)	24 (4898)
Other medical specialties (*n* = 197)	22 (10.78)	16 (7.84)	23 (11.27)	18 (8.82)	125 (61.27)
Total (*n* = 424)	47 (11.08)	44 (10.38)	49 (11.56)	34 (8.02)	250 (58.96)
Dentists					
Paediatric dentists (*n* = 26)	4 (15.38)	6 (23.08)	5 (19.23)	2 (7.69)	9 (34.62)
Orthodontists (*n* = 96)	20 (20.83)	10 (10.42)	20 (20.83)	11 (11.46)	35 (36.46)
Oral surgeons (*n* = 39)	2 (5.13)	3 (7.69)	7 (17.95)	4 (10.26)	23 (58.97)
Other dental specialties (*n* = 258)	40 (15.50)	26 (10.08)	28 (10.85)	25 (9.69)	139 (53.88)
Total (*n* = 419)	66 (15.75)	45 (10.74)	60 (14.32)	42 (10.02)	206 (49.16)

**Fig. 1:**
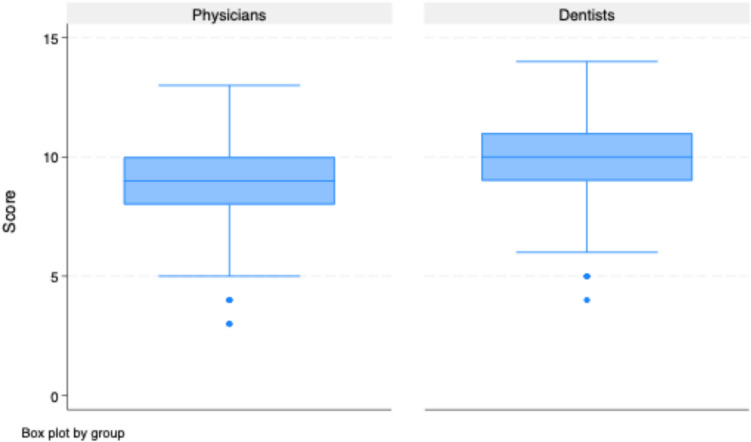
Box plot illustrating the distribution of total questionnaire scores of physicians and dentists

The comparison between physicians and dentists reveals notable differences in knowledge and attitudes related to paediatric dentistry, as reflected in their questionnaire responses. Overall, dentists achieved significantly higher scores than physicians, providing strong evidence of a difference (*p* < 0.01) (Fig. 1 and Table 2).

**Table 2:** Comparison of correct response rates to questionnaire items between physicians and dentists

Score	Physicians	Dentists	*p* value
	*n* = 424	*n* = 419	
	mean (± SD)	mean (± SD)	
Knowledge items	8.64 (± 1.83)	9.78 (± 1.81)	< 0.01
	% (*n*)	% (*n*)	
At what age do the first primary teeth erupt?	95.52 (405)	98.57 (413)	0.02
How many primary teeth are there?	81.13 (344)	99.52 (417)	< 0.01
At what age do the last primary teeth change?	70.28 (298)	76.85 (322)	0.04
Can a high concentration of cariogenic bacteria in the mother facilitate early colonisation of the child’s oral cavity?	66.98 (284)	79.00 (331)	< 0.01
Is systemic fluoride administration more effective than topical administration?	62.03 (263)	69.45 (261)	0.03
Is sealing the first permanent molar an effective method of caries prevention?	82.08 (348)	96.90 (406)	< 0.01
Does pacifier use appropriate in a 4 year-old child?	94.34 (400)	95.94 (402)	0.36
Can an initial caries lesion (or “white spot lesion”) be reversed?	86.79 (368)	66.83 (280)	< 0.01
Is it necessary to intervene to stop bruxism in children?	28.30 (120)	76.84 (322)	< 0.01
Attitudes items			
At what age is it recommended for children to visit the dentist for the first time?	16.27 (69)	30.55 (128)	< 0.01
Are toothpastes with 500 ppm fluoride recommended for children of all ages?	59.91 (254)	57.28 (240)	0.48
How much toothpaste should be used for a child aged 6 months to 3 years?	27.59 (117)	38.19 (160)	< 0.01
At what age is it recommended for children to stop requiring parental supervision for tooth brushing?	71.70 (304)	73.99 (310)	0.50
What is the correct advice to give parents in the event of dental trauma involving the avulsion of a permanent tooth?	21.23 (90)	17.66 (74)	0.22
Up to what age should breastfeeding be recommended?*			
Up to 1 year of age	221 (52.12)	275 (65.63)	< 0.01
Up to 2 years of age	81 (19.10)	85 (20.29)	
After 2 years of age, for as long as the mother deems it necessary or beneficial	122 (28.77)	59 (14.08)	

^*^Item with no correct answer

Dentists demonstrated superior knowledge on several topics, including the total number of primary teeth (99.52% correct responses vs. 81.13% for physicians, with strong evidence of a difference, *p* < 0.01), the transmissibility of cariogenic bacteria from mother to child (79.0% vs. 66.98%, with strong evidence of a difference, *p* < 0.01), the effectiveness of sealants in preventing caries in permanent first molars (96.9% vs. 82.08%, with robust evidence of a difference, *p* < 0.01), and the lack of necessity to intervene to stop bruxism in children (76.84% vs. 28.30% with very strong evidence of a difference, *p* < 0.01). In contrast, physicians outperformed dentists in recognising that initial caries lesions can be reversed (86.79% vs. 66.83%, with strong evidence of a difference, *p* < 0.01).

In addition to statistical significance, the magnitude of the difference between groups was assessed using Cohen’s *d*. The difference in total score between dentists and physicians (mean difference = 1.14 points) corresponded to a Cohen’s d of 0.63, indicating a medium-to-large effect size. This suggests that the observed difference is not only statistically significant but also educationally meaningful. For selected categorical comparisons, effect sizes were calculated using Cramér’s *V*. The difference in correct responses regarding the number of primary teeth yielded a Cramér’s *V* of 0.34 (moderate effect). The difference concerning the recommended age for the first dental visit showed a smaller effect (Cramér’s *V* = 0.17), indicating that whilst statistically significant, the practical magnitude of this difference was more limited.

In terms of attitudes, physicians appeared to demonstrate a higher level of comprehension, albeit not statistically significant, than dentists with respect to the incompatibility of toothpaste containing 500 ppm of fluoride with children of all ages (59.91% vs. 57.28%, *p* = 0.48, with no evidence of a difference). Nevertheless, both groups exhibited significant gaps. For instance, only 16.27% of physicians and 30.55% of dentists correctly identified the appropriate age for a first dental visit, with robust evidence of a difference (*p* < 0.01). Furthermore, 21.23% of physicians and 17.66% of dentists knew the correct advice to give parents in case of dental trauma (Table 2).

**Fig. 2:**
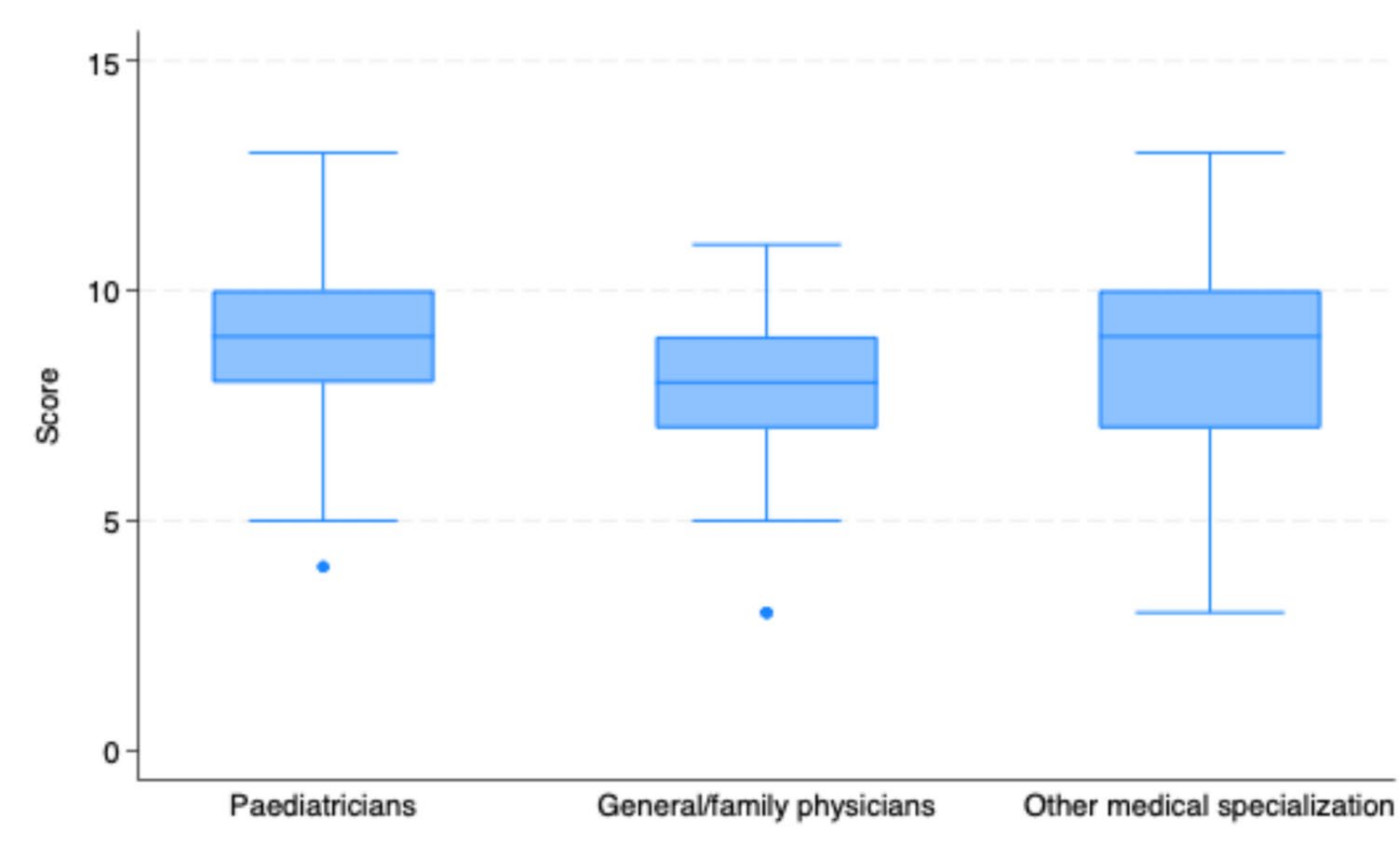
Box plot illustrating the distribution of total questionnaire scores across physician specialties (paediatricians, general/family physicians, and other medical specialties)

ANOVA was used to assess differences within each group based on the different specialties, revealing substantial variations among paediatricians, general/family physicians, and other medical specialists in mean scores, with the assumption of equal variances being met. Amongst physicians, the ANOVA produced a significant *F*-statistic of 9.59 (*p* < 0.01), strongly indicating differences across subgroups. Bartlett’s test confirmed the assumption of equal variances (*p* = 0.41) (Fig. 2). Differences between each subgroup were found with Tukey’s test (Supplementary file S3).

**Fig. 3:**
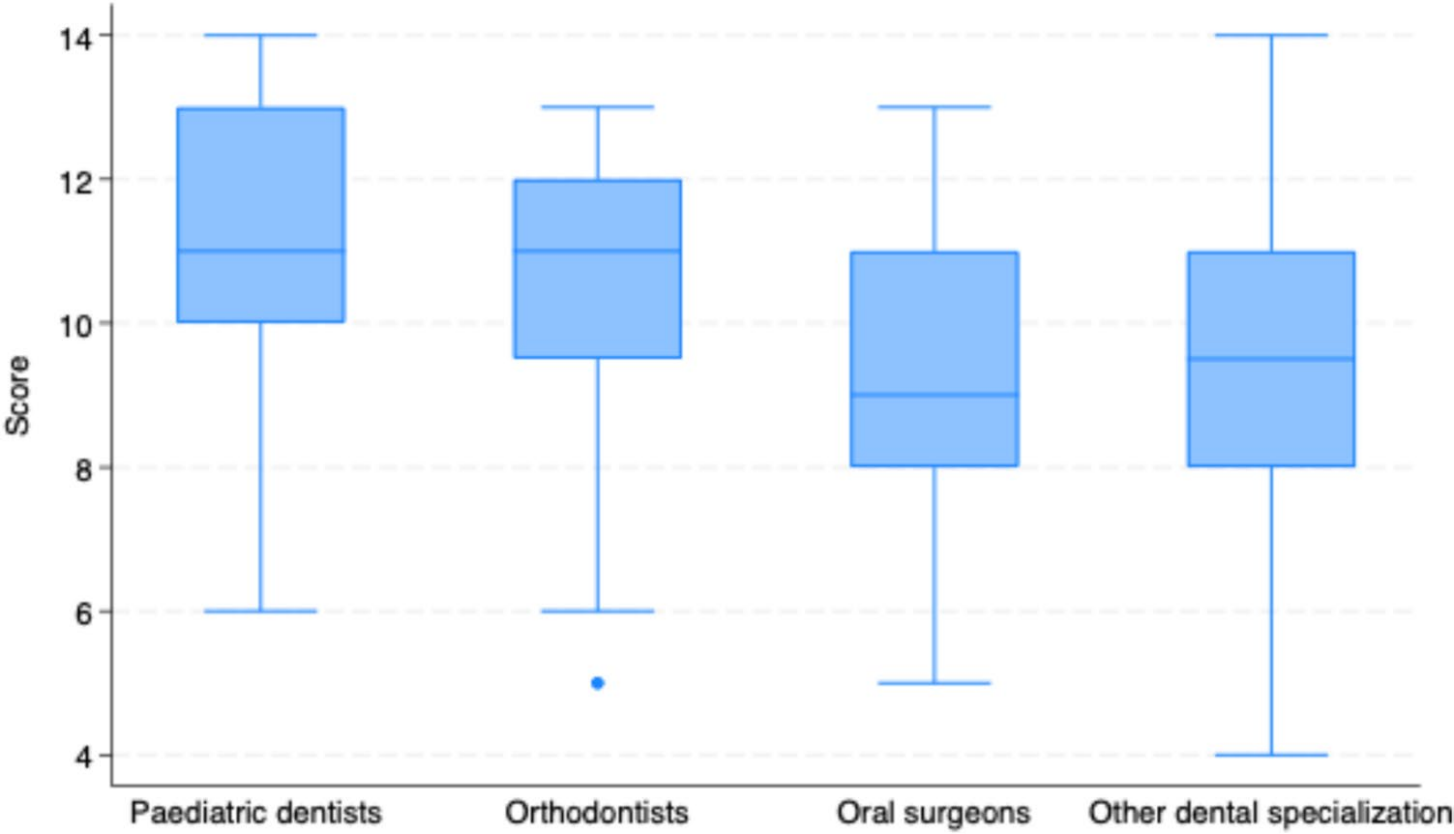
Box plot illustrating the distribution of total questionnaire scores across dentists with different specialties (paediatric dentists, orthodontics, oral surgeons and other dental specialisations)

Amongst dentists, the *F*-statistic was even higher (12.40, *p* < 0.01), providing very strong evidence of differences in score amongst paediatric dentists, orthodontists, oral surgeons and other dental specialists. Bartlett’s test also supported the assumption of equal variances in this group (*p* = 0.09) (Fig. 3). Tukey’s test revealed significant differences between subgroups, except for the comparisons between paediatric dentists and orthodontists, as well as between oral surgeons and other dental specialists (Supplementary file S3).

The linear regression analysis (Table 3) showed that both predictors (group and years of working experience) were able to contribute to explaining the score variable with strong evidence. Belonging to the dentist group was associated with an average increase of 1.08 points in the questionnaire score compared to the physician group in subjects with the same years of working experience. On the other hand, years of working experience were negatively associated with the score. Specifically, a 1-unit increase among the categories of years of work experience was associated with an average decrease of 0.19 in the variable score among subjects of the same group.

**Table 3:** Linear regression analysis assessing the impact of professional group and years of working experience on total questionnaire score among healthcare professionals

	Coefficient	SE	*t*	*p*	95%CI
Group	1.08	0.12	8.69	< 0.01	0.84, 1.33
Years of working experience	− 1.89	0.04	− 4.56	< 0.01	− 0.27, − 0.11
Intercept	9.38	0.18	50.7	< 0.01	9.02, 9.75

*F*-statistic = 52.22; *p* < 0.01; *R* = 0.11 adjusted *R*^2^ = 0.11

## Discussion

The present study focuses on the knowledge and attitudes of health professionals regarding paediatric dentistry, revealing disparities between physicians and dentists and highlighting gaps and inconsistencies in understanding and applying key oral health concepts. Importantly, some of these discrepancies should be interpreted considering the heterogeneity of national and international guidelines, which may contribute to variability in clinical responses rather than reflecting an unequivocal lack of knowledge and attitudes.

The results indicate differences between physicians’ and dentists’ preparedness level, with dentists outperforming physicians on most topics, but the gap was smaller than expected. Dentists demonstrated a stronger grasp of the number of primary teeth, the colonisation of a child’s mouth by cariogenic bacteria from the mother, and the effectiveness of sealants in caries prevention, likely reflecting their specialised training. However, physicians exhibited superior knowledge in certain areas, such as recognising the reversibility of initial carious lesions. Regarding attitudes, physicians appeared to have a better understanding of the appropriate fluoride concentration in children’s toothpaste; however, they were less able to recommend the correct amount of toothpaste for children under 3 years of age. Additionally, both specialists demonstrated limited ability to provide parents with correct and timely advice in the event of dental trauma.

The ongoing confusion surrounding fluoride use in children is particularly noteworthy, considering that Italian and European guidelines consistently recommend toothpaste containing at least 1000 ppm fluoride for children up to 6 years of age, and 1450 ppm thereafter (Italian guidelines, Minister of Health 2013; Toumba et al. 2019; AAPD 2023). Whilst it is acknowledged that recommendations on fluoride use may vary across countries and professional organisations outside Italy and the European Union, the present study was conducted exclusively within the Italian context. Therefore, a higher level of adherence to national guidelines would reasonably be expected among Italian healthcare professionals. Despite this, discrepancies in clinical behaviours, confirmed by the study results, remain evident and merit further exploration. Notably, a study in Alabama reported that many physicians recommended the introduction of fluoride-containing toothpaste between 1 and 3 years of age (Sánchez et al. 1997), and in Catalonia only a minority of paediatricians recognised the importance of using toothpaste with a minimum fluoride concentration of 1000 ppm (Morera-Domingo et al. 2022). Whilst a survey conducted in Italy nearly two decades ago revealed that almost all paediatricians reported prescribing fluoride supplementation, the present study indicates that most dentists and physicians attribute greater value to topical fluoride in caries prevention compared to systemic fluoride administration (Di Giuseppe et al. 2006). These findings underscore not only the variation in clinical guidelines amongst countries and professional communities, but also a potentially shifting perspective on optimal fluoride use in paediatric care. The Italian recommendation to use a very small amount of fluoridated toothpaste, comparable to a grain of rice, for children aged 6 months to 3 years appears to be insufficiently familiar to a substantial proportion of both dentists and physicians in the present sample. This recommendation appears to be unfamiliar to most of the sample; proper dosing of fluoride toothpaste is essential to prevent caries while minimising the risk of developing dental fluorosis (Wong et al. 2024).

Cariogenic bacteria colonise a child’s oral cavity through saliva sharing when teeth start erupting (Law et al. 2007). The early presence of cariogenic bacteria, particularly the mutans streptococci group, in combination with a diet rich in fermentable carbohydrates, constitutes a significant risk factor for caries lesion development. Elevated levels of these bacteria in the mother’s saliva significantly increase the likelihood of early colonisation in the child’s mouth (Childers et al. 2017; Colombo et al. 2019). Although in the present survey most participants were aware of the early colonisation of cariogenic bacteria, this information is less known amongst physicians. As a result, they may fail to provide expectant or new mothers with valuable guidance to improve their own oral health behaviours, safeguarding the health of their children.

Dental sealants have been proven to be effective for caries prevention and the management of initial caries lesions of pits and fissures of molars, vulnerable areas to caries in young children (Balian et al. 2022; Leite et al. 2024). The effectiveness of this preventive strategy appears to be well known amongst dentists, but not as much amongst physicians. Conversely, it is surprising that more dentists than physicians are unaware of the possibility that an initial carious lesion can be reversed with appropriate non-operative therapy. This gap reflects a treatment approach of dentists more oriented towards restorative dentistry than towards non-invasive caries lesion management (Cagetti & Angelino 2021).

Although an early dental visit is crucial for establishing preventive care and identifying risk factors in the newborn, findings of the present survey show some confusion among responders, as it was reported amongst general dentists in Michigan (Clark et al. 2014). Responses regarding the timing of a child’s first dental visit should be interpreted with caution, as guidelines vary internationally. Whilst organisations such as the EAPD and AAPD recommend the first dental visit within the first year of life (EAPD 1997; AAPD 2023), Italian national guidelines do not provide a specific age recommendation (Italian Minister of Health 2013). Thus, variability in responses may reflect differences in guideline interpretation rather than a lack of professional attitude. Furthermore, the questionnaire used in this study was based on considering age 3 as the benchmark for the first dental visit; therefore, this item was not modified in the present survey.

Inadequate attitudes regarding the management of dental trauma were also observed in the present sample, emphasising the need for targeted educational efforts, as improper handling can result in severe complications. Similar results were reported in Croatia, where one-fifth of respondents believed that a permanent tooth should not be reimplanted (Nikolic et al. 2018).

The appropriate age to discontinue breastfeeding is a particularly heterogeneous topic. Guidelines from paediatric and paediatric dentistry organisations are not entirely consistent, which likely contributes to the observed variability in recommendations given to mothers (AAPD 2008; Meek and Noble 2022). The results of the present survey highlight this ambivalence—about half of the physicians recommend breastfeeding until the age of 2 years or even longer as the mother considers it beneficial, whereas only one-third of the dentists provide a similar recommendation. Whilst breastfeeding should always be encouraged, its prolonged use, particularly on demand during the night, can be a risk factor for caries lesion development if not followed by proper oral hygiene practices (Cagetti et al. 2024). Shared recommendations between paediatric specialists could help overcome this impasse and resolve the ambiguities surrounding the issue.

Subgroup analysis offers valuable insights. Amongst physicians, paediatricians demonstrated the highest level of preparedness, likely due to their specialised focus on child health. In the dental field, paediatric dentists and orthodontists demonstrated higher levels of expertise. However, the considerable variability observed within each subgroup suggests uneven training and exposure, even amongst specialists within the same discipline. This reflects a lack of standardisation in curricula and emphasises the need for more uniform education and training across institutions. Paediatricians’ knowledge was found to be poor, and their participation in oral health continues to be limited in different countries. The potential for the non-dental workforce to greatly improve children’s oral health is underexploited (Farsi and Alagili 2023; Jafari et al. 2023).

Although the regression analysis identified statistically significant associations between professional group, years of working experience, and total preparedness score, the overall explained variance of the model was modest. This finding may indicate that a substantial proportion of variability in knowledge and attitudes towards paediatric oral health remains unexplained by the variables included in the model. This relatively low explanatory power suggests that additional factors not assessed in the present study, such as participation in continuing education programmes, exposure to updated clinical guidelines, workplace setting, interdisciplinary collaboration, and personal professional interest, may substantially influence preparedness levels (Lee 2023). Therefore, the regression results should be interpreted as identifying relevant but partial predictors, rather than providing a comprehensive explanation of determinants of professional competence in paediatric dentistry.

The findings of the present study should be interpreted considering several limitations. Despite the relatively large absolute number of respondents, the low response rate observed in the present study represents an important limitation that warrants careful consideration. A response rate of just over 3% raises concerns regarding potential selection bias, as it is plausible that health professionals with a greater interest or awareness of paediatric oral health were more likely to participate. Consequently, the overall level of knowledge and attitudes observed among both physicians and dentists may be overestimated relative to the broader professional population. This limitation may also affect the generalisability of the findings, particularly in comparisons across professional subgroups and specialties, as the responding sample may not be fully representative of all physicians and dentists practising in different clinical contexts. Nevertheless, the consistency of the observed patterns, the internal coherence of the responses, and the alignment with existing literature (Romano and Silk 2023) support the relevance of the identified preparedness gaps and interprofessional differences. The cross-sectional design limits causal inference. Whilst associations between professional group, years of experience, and total preparedness score were identified, the temporal direction of these relationships cannot be established. Although the questionnaire underwent content validation and pre-testing for comprehensibility, it was not designed as a psychometric scale measuring a single latent construct. The items covered multiple domains of paediatric oral health (e.g. eruption timing, fluoride use, trauma management, sealants, and breastfeeding recommendations), which may limit the internal coherence of the total score. Whilst the aggregation of items provided a global indicator of overall preparedness, the multidimensional nature of the instrument may introduce heterogeneity that is not fully captured by a single composite score. Moreover, the survey relied on self-administered responses, which may be subject to response bias. Participants may have consulted external sources while completing the questionnaire or provided answers perceived as socially desirable. Additionally, the online format does not allow verification of independent completion. Moreover, some items addressed topics for which international and national guidelines are not fully harmonised (e.g. duration of breastfeeding, timing of the first dental visit). In these cases, variability in responses may reflect differences in guideline interpretation or professional perspective rather than unequivocal knowledge deficits. Therefore, certain discrepancies observed between professional groups should be interpreted with caution.

Despite these limitations, the study provides valuable exploratory insights into interprofessional differences and highlights areas where targeted educational interventions and guideline harmonisation may improve paediatric oral healthcare delivery.

The results of this survey underscore the critical importance of lifelong learning and continuing education programmes to ensure professionals remain updated on current guidelines and evidence-based practices (Lee 2023). Greater integration of oral health topics into medical education, particularly for general practitioners and paediatricians, is essential to ensure consistent messaging and early intervention. Similarly, strengthening continuing education for dentists could significantly enhance the quality of paediatric oral healthcare. Efforts should also be made to harmonise clinical guidelines across national and international organisations to provide clear and consistent recommendations for professionals. Expanding the sample could provide a broader understanding of these issues. Future research should also focus on evaluating the long-term impact of educational interventions and identifying strategies to sustain improvements in knowledge and practices.

Targeted continuing education initiatives, particularly in areas such as fluoride use, trauma management, early dental visits, and non-invasive caries management, may contribute to more consistent preventive messaging and improved paediatric oral healthcare delivery.

Future research with higher response rates, broader geographic representation, and longitudinal designs is needed to confirm these findings and to identify determinants of professional competence more comprehensively. Evaluating the impact of structured educational interventions on knowledge retention and clinical practice would also represent an important next step.

## Conclusions

Within the limits of this cross-sectional survey and considering the low response rate, the findings suggest that differences in knowledge and clinical attitudes towards paediatric oral health exist between dentists and physicians in this sample from Northern Italy. Dentists achieved higher overall scores than physicians, with a medium-to-large magnitude of difference; however, relevant gaps and areas of uncertainty were observed in both professional groups. Importantly, variability was also evident within subgroups of the same discipline, indicating that professional specialisation alone does not guarantee consistent preparedness in paediatric oral health. Paediatricians, paediatric dentists, and orthodontists tended to demonstrate higher levels of knowledge and attitudes compared to other specialties, yet heterogeneity persisted even among these subgroups. These findings highlight the influence of training pathways, continuing education exposure, and possibly generational differences in curricula. The study underscores the importance of strengthening interdisciplinary collaboration, integrating paediatric oral health more consistently into medical and dental curricula, and promoting harmonised, evidence-based clinical guidelines.

## Supplementary Information

Below is the link to the electronic supplementary material.

Supplementary file1 (DOCX 44 KB)

## Data Availability

The datasets used and analysed during the current study are available from the corresponding author on reasonable request.
