# Supplementary material for: Children’s oral health: Are dentists and physicians prepared enough? A cross-sectional interdisciplinary survey carried out in North Italy
Source: Eur Arch Paediatr Dent. 2026 Apr 1;27(4):1221–31. doi: 10.1007/s40368-026-01203-0 (PMC13461947; doi:10.1007/s40368-026-01203-0)
Supplement: Supplementary file 1 [file 40368_2026_1203_MOESM1_ESM.docx]

**Children's oral health: are dentists and physicians enough prepared? An Interdisciplinary Survey carried out in North Italy.**

**Maria Grazia Cagetti, Claudia Salerno, Silvia Cirio, Guglielmo Campus^3^**

Supplementary file

S1. Study questionnaire

S2. Content Validity Index and Content Validity Ratio

S3. Variables re-coding in numerical score

S4. Post-hoc Tukey test

S1. Study questionnaire

1. **Specialization**
a. Pediatricians
b. General/family physicians
c. Other medical specializations
d. Pediatric dentists
e. Orthodontists
f. Oral surgeons
g. Other dental specializations
2. **Working experience**
   a. < 5 years
   b. 5-10 years
   c. 11-15 years
   d. 16-20 years
   e. > 20 years
3. **At what age do the first primary teeth erupt?**
a. 6-12 months
b. > 12 months
4. **How** **many deciduous teeth are there?**
   a. 16
   b. 18
   c. 20
   d. 30
5. **At what age do the last deciduous teeth change?**
   a. 0-3 years
   b. 6-7 years
   c. 8-9 years
   d. 10-12 years
6. **Can a high concentration of cariogenic bacteria in the mother facilitate early colonization of the child's oral cavity?**
   a. Yes
   b. No
7. **At what age is it recommended for children to visit the dentist for the first time?**
   a. Up to 3 years of age
   b. Between 3- and 6-years of age
   c. After 6 years of age, when the change of primary teeth starts
   d. When all permanent teeth changed
8. **Are toothpastes with 500 ppm fluoride recommended for children of all ages?**
   a. Yes
   b. No
9. **Is systemic fluoride administration more effective than topical administration?**
   a. Yes
   b. No
10. **Up to what age should breastfeeding be recommended?**
    a. Up to 1 year of age
    b. Up to 2 years of age
    c. After 2 years of age, for as long as the mother deems it necessary or beneficial
11. **At what age is it recommended for children to stop requiring parental supervision for tooth brushing?**
    a. Up to 1 year of age
    b. Up to 3 years of age
    c. Up to 6 years of age
    d. Until the child developed good manual skills
12. **How much toothpaste should be used for a child aged 6 months to 3 years?**
    a. Rice-size
    b. Pea-size
    c. It should be completely covered the toothbrush
    d. The amount is not important
    e. It shouldn’t be using toothpaste for this age children
13. **Does pacifier use appropriate in a 4-year-old child?**
    a. Yes
    b. No
14. **Can an initial caries lesion (or “white spot lesion”) be reversed?**
    a. Yes
    b. No
15. **Is sealing the first permanent molar an effective method of caries prevention?**
    a. Yes
    b. No
16. **Is it necessary to intervene to stop bruxism in children?**
    a. Yes
    b. No
17. **What is the correct advice to give parents in the event of dental trauma involving the avulsion of a permanent tooth?**
    a. Keep the tooth in saliva, milk or water and go to the dentist as soon as possible
    b. Go to the emergency room
    c. Rince the tooth, take it from the crown and put it in the dental alveolus; then, go to the dentist as soon as possible
    d. Stop the bleeding and go to the dentist even the day after, because the tooth can’t be reimplanted

S2. Content Validity Index and Content Validity Ratio

|  | **Item** | **VCI(R)** | **VCI(C)** | **VCI** | **CVR** |
| --- | --- | --- | --- | --- | --- |
| 1 | **Specialization** |  |  |  |  |
|  | Pediatricians | 1.00 | 1.00 | 1.00 | 1.00 |
|  | General/family physicians | 1.00 | 1.00 | 1.00 | 1.00 |
|  | Other medical specializations | 1.00 | 1.00 | 1.00 | 1.00 |
|  | Pediatric dentists | 1.00 | 1.00 | 1.00 | 1.00 |
|  | Orthodontists | 1.00 | 1.00 | 1.00 | 1.00 |
|  | Oral surgeons | 1.00 | 1.00 | 1.00 | 1.00 |
|  | Other dental specializations | 1.00 | 1.00 | 1.00 | 1.00 |
| 2 | **Working experience** |  |  |  |  |
|  | < 5 years | 1.00 | 1.00 | 1.00 | 1.00 |
|  | 5-10 years | 1.00 | 1.00 | 1.00 | 1.00 |
|  | 11-15 years | 1.00 | 1.00 | 1.00 | 1.00 |
|  | 16-20 years | 1.00 | 1.00 | 1.00 | 1.00 |
|  | > 20 years | 1.00 | 1.00 | 1.00 | 1.00 |
| 3 | **At what age do the first primary teeth erupt?** |  |  |  |  |
|  | 6-12 months | 1.00 | 1.00 | 1.00 | 1.00 |
|  | > 12 months | 1.00 | 1.00 | 1.00 | 1.00 |
| 4 | **How** **many deciduous teeth are there?** |  |  |  |  |
|  | 16 | 1.00 | 1.00 | 1.00 | 1.00 |
|  | 18 | 1.00 | 1.00 | 1.00 | 1.00 |
|  | 20 | 1.00 | 1.00 | 1.00 | 1.00 |
|  | 30 | 1.00 | 1.00 | 1.00 | 1.00 |
| 5 | **At what age do the last deciduous teeth change?** |  |  |  |  |
|  | 0-3 years | 1.00 | 1.00 | 1.00 | 0.80 |
|  | 6-7 years | 1.00 | 1.00 | 1.00 | 0.80 |
|  | 8-9 years | 1.00 | 1.00 | 1.00 | 0.80 |
|  | 10-12 years | 1.00 | 1.00 | 1.00 | 0.80 |
| 6 | **Can a high concentration of cariogenic bacteria in the mother facilitate early colonization of the child's oral cavity?** |  |  |  |  |
|  | Yes | 1.00 | 1.00 | 1.00 | 1.00 |
|  | No | 1.00 | 1.00 | 1.00 | 1.00 |
| 7 | **At what age is it recommended for children to visit the dentist for the first time?** |  |  |  |  |
|  | Up to 3 years of age | 1.00 | 1.00 | 1.00 | 1.00 |
|  | Between 3- and 6-years of age | 1.00 | 1.00 | 1.00 | 1.00 |
|  | After 6 years of age, when the change of primary teeth starts | 1.00 | 1.00 | 1.00 | 1.00 |
|  | When all permanent teeth changed | 1.00 | 1.00 | 1.00 | 1.00 |
| 8 | **Are toothpastes with 500 ppm fluoride recommended for children of all ages?** |  |  |  |  |
|  | Yes | 1.00 | 1.00 | 1.00 | 1.00 |
|  | No | 1.00 | 1.00 | 1.00 | 1.00 |
| 9 | **Is systemic fluoride administration more effective than topical administration?** |  |  |  |  |
|  | Yes | 1.00 | 1.00 | 1.00 | 1.00 |
|  | No | 1.00 | 1.00 | 1.00 | 1.00 |
| 10 | **Up to what age should breastfeeding be recommended?** |  |  |  |  |
|  | Up to 1 year of age | 1.00 | 1.00 | 1.00 | 1.00 |
|  | Up to 2 years of age | 1.00 | 1.00 | 1.00 | 1.00 |
|  | After 2 years of age, for as long as the mother deems it necessary or beneficial | 1.00 | 1.00 | 1.00 | 1.00 |
| 11 | **At what age is it recommended for children to stop requiring parental supervision for tooth brushing?** |  |  |  |  |
|  | Up to 1 year of age | 1.00 | 1.00 | 1.00 | 1.00 |
|  | Up to 3 years of age | 1.00 | 1.00 | 1.00 | 1.00 |
|  | Up to 6 years of age | 1.00 | 1.00 | 1.00 | 1.00 |
|  | Until the child developed good manual skills | 1.00 | 1.00 | 1.00 | 1.00 |
| 12 | **How much toothpaste should be used for a child aged 6 months to 3 years?** |  |  |  |  |
|  | Rice-size | 1.00 | 1.00 | 1.00 | 1.00 |
|  | Pea-size | 1.00 | 1.00 | 1.00 | 1.00 |
|  | It should be completely covered the toothbrush | 1.00 | 1.00 | 1.00 | 1.00 |
|  | The amount is not important | 1.00 | 1.00 | 1.00 | 1.00 |
|  | It shouldn’t be using toothpaste for this age children | 1.00 | 1.00 | 1.00 | 1.00 |
| 13 | **Does pacifier use appropriate in a 4-year-old child?** |  |  |  |  |
|  | Yes | 1.00 | 1.00 | 1.00 | 1.00 |
|  | No | 1.00 | 1.00 | 1.00 | 1.00 |
| 14 | **Can an initial caries lesion (or “white spot lesion”) be reversed?** |  |  |  |  |
|  | Yes | 1.00 | 1.00 | 1.00 | 1.00 |
|  | No | 1.00 | 1.00 | 1.00 | 1.00 |
| 15 | **Is sealing the first permanent molar an effective method of caries prevention?** |  |  |  |  |
|  | Yes | 1.00 | 1.00 | 1.00 | 1.00 |
|  | No | 1.00 | 1.00 | 1.00 | 1.00 |
| 16 | **Is it necessary to intervene to stop bruxism in children?** |  |  |  |  |
|  | Yes | 1.00 | 1.00 | 1.00 | 1.00 |
|  | No | 1.00 | 1.00 | 1.00 | 1.00 |
| 17 | **What is the correct advice to give parents in the event of dental trauma involving the avulsion of a permanent tooth?** |  |  |  |  |
|  | Keep the tooth in saliva, milk or water and go to the dentist as soon as possible | 1.00 | 1.00 | 1.00 | 1.00 |
|  | Go to the emergency room | 1.00 | 1.00 | 1.00 | 1.00 |
|  | Rince the tooth, take it from the crown and put it in the dental alveolus; then, go to the dentist as soon as possible | 1.00 | 1.00 | 1.00 | 1.00 |
|  | Stop the bleeding and go to the dentist even the day after, because the tooth can’t be reimplanted | 1.00 | 1.00 | 1.00 | 1.00 |
|  | **Overall** | **1.00** | **1.00** | **1.00** | **1.00** |
|  | CVI: Content Validity Index; CVR: Content Validity Ratio; R: Relevancy; C: Clarity | | | | |

S3. Variables re-coding in numerical score

|  | **Item** | **Score** |
| --- | --- | --- |
| 1 | **Specialization** |  |
|  | Pediatricians | n.a. |
|  | General/family physicians | n.a. |
|  | Other medical specializations | n.a. |
|  | Pediatric dentists | n.a. |
|  | Orthodontists | n.a. |
|  | Oral surgeons | n.a. |
|  | Other dental specializations | n.a. |
| 2 | **Working experience** |  |
|  | < 5 years | n.a. |
|  | 5-10 years | n.a. |
|  | 11-15 years | n.a. |
|  | 16-20 years | n.a. |
|  | > 20 years | n.a. |
| 3 | **At what age do the first primary teeth erupt?** |  |
|  | 6-12 months | 1 |
|  | > 12 months | 0 |
| 4 | **How** **many deciduous teeth are there?** |  |
|  | 16 | 0 |
|  | 18 | 0 |
|  | 20 | 1 |
|  | 30 | 0 |
| 5 | **At what age do the last deciduous teeth change?** |  |
|  | 0-3 years | 0 |
|  | 6-7 years | 0 |
|  | 8-9 years | 0 |
|  | 10-12 years | 1 |
| 6 | **Can a high concentration of cariogenic bacteria in the mother facilitate early colonization of the child's oral cavity?** |  |
|  | Yes | 1 |
|  | No | 0 |
| 7 | **At what age is it recommended for children to visit the dentist for the first time?** |  |
|  | Up to 3 years of age | 1 |
|  | Between 3- and 6-years of age | 0 |
|  | After 6 years of age, when the change of primary teeth starts | 0 |
|  | When all permanent teeth changed | 0 |
| 8 | **Are toothpastes with 500 ppm fluoride recommended for children of all ages?** |  |
|  | Yes | 0 |
|  | No | 1 |
| 9 | **Is systemic fluoride administration more effective than topical administration?** |  |
|  | Yes | 0 |
|  | No | 1 |
| 10 | **Up to what age should breastfeeding be recommended?** |  |
|  | Up to 1 year of age | n.a. |
|  | Up to 2 years of age | n.a. |
|  | After 2 years of age, for as long as the mother deems it necessary or beneficial | n.a. |
| 11 | **At what age is it recommended for children to stop requiring parental supervision for tooth brushing?** |  |
|  | Up to 1 year of age | 0 |
|  | Up to 3 years of age | 0 |
|  | Up to 6 years of age | 0 |
|  | Until the child developed good manual skills | 1 |
| 12 | **How much toothpaste should be used for a child aged 6 months to 3 years?** |  |
|  | Rice-size | 1 |
|  | Pea-size | 0 |
|  | It should be completely covered the toothbrush | 0 |
|  | The amount is not important | 0 |
|  | It shouldn’t be using toothpaste for this age children | 0 |
| 13 | **Does pacifier use appropriate in a 4-year-old child?** |  |
|  | Yes | 0 |
|  | No | 1 |
| 14 | **Can an initial caries lesion (or “white spot lesion”) be reversed?** |  |
|  | Yes | 1 |
|  | No | 0 |
| 15 | **Is sealing the first permanent molar an effective method of caries prevention?** |  |
|  | Yes | 1 |
|  | No | 0 |
| 16 | **Is it necessary to intervene to stop bruxism in children?** |  |
|  | Yes | 0 |
|  | No | 1 |
| 17 | **What is the correct advice to give parents in the event of dental trauma involving the avulsion of a permanent tooth?** |  |
|  | Keep the tooth in saliva, milk or water and go to the dentist as soon as possible | 0 |
|  | Go to the emergency room | 0 |
|  | Rince the tooth, take it from the crown and put it in the dental alveolus; then, go to the dentist as soon as possible | 1 |
|  | Stop the bleeding and go to the dentist even the day after, because the tooth can’t be reimplanted | 0 |
| n.a.: not applicable | | |

S4. Post-hoc Tukey test

|  | Contrast | Standard error | t | p |
| --- | --- | --- | --- | --- |
| Pediatricians *vs* General/family physicians | -1.20 | 0.29 | -4.11 | 0.00 |
| Pediatricians *vs* Other medical specializations | -0.53 | 0.19 | -2.84 | 0.01 |
| Other medical specializations *vs* General/family physicians | 0.67 | 0.29 | 2.34 | 0.05 |
| Pediatric dentists *vs* Orthodontists | -0.53 | 0.38 | -1.39 | 0.51 |
| Pediatric dentists *vs* Oral surgeons | -1.86 | 0.44 | -4.22 | 0.00 |
| Pediatric dentists *vs* Other dental specializations | -1.45 | 0.36 | -4.04 | 0.00 |
| Orthodontists *vs* Oral surgeons | -1.32 | 0.33 | -4.01 | 0.00 |
| Orthodontists *vs* Other dental specializations | -0.91 | 0.21 | -4.39 | 0.00 |
| Oral surgeons *vs* Other dental specializations | 0.41 | 0.30 | 1.38 | 0.51 |
